# Absolute lung size and the sex difference in breathlessness in the general population

**DOI:** 10.1371/journal.pone.0190876

**Published:** 2018-01-05

**Authors:** Magnus Ekström, Josefin Sundh, Linus Schiöler, Eva Lindberg, Annika Rosengren, Göran Bergström, Oskar Angerås, Jan Hedner, John Brandberg, Björn Bake, Kjell Torén

**Affiliations:** 1 Department of Respiratory Medicine and Allergology, Institution for Clinical Sciences, Lund University, Lund, Sweden; 2 Department of Respiratory Medicine, School of Medical Sciences, Örebro University, Örebro, Sweden; 3 Section of Occupational and environmental medicine, Sahlgrenska Academy, University of Gothenburg, Gothenburg, Sweden; 4 Department of Medical Sciences, Respiratory-, Allergy- and Sleep Research, Uppsala University, Uppsala, Sweden; 5 The Wallenberg Laboratory, Department of Molecular and Clinical Medicine, Institute of Medicine, Sahlgrenska University Hospital, University of Gothenburg, Gothenburg, Sweden; 6 Department of Cardiology, Sahlgrenska University Hospital, Gothenburg, Sweden; 7 Department of Internal Medicine, Institute of Medicine, University of Gothenburg, Gothenburg, Sweden; 8 Department of Radiology, Institute of Clinical Sciences, University of Gothenburg, Gothenburg, Sweden; 9 Department of Respiratory Medicine and Allergology, University of Gothenburg, Gothenburg, Sweden; Lee Kong Chian School of Medicine, SINGAPORE

## Abstract

**Background:**

Breathlessness is associated with major adverse health outcomes and is twice as common in women as men in the general population. We evaluated whether this is related to their lower absolute lung volumes.

**Methods:**

Cross-sectional analysis of the population-based Swedish CardioPulmonarybioImage Study (SCAPIS) Pilot, including static spirometry and diffusing capacity (n = 1,013; 49% women). Breathlessness was measured using the modified Medical Research Council (mMRC) scale and analyzed using ordinal logistic regression adjusting for age, pack-years of smoking, body mass index, chronic airway limitation, asthma, chronic bronchitis, depression and anxiety in all models.

**Results:**

Breathlessness was twice as common in women as in men; adjusted odds ratio (OR) 2.20 (95% confidence interval, 1.32−3.66). Lower absolute lung volumes were associated with increased breathlessness prevalence in both men and women. The sex difference in breathlessness was unchanged when adjusting for lung function in %predicted, but disappeared when controlling for *absolute values* of total lung capacity (OR 1.12; 0.59−2.15), inspiratory capacity (OR 1.26; 0.68−2.35), forced vital capacity (OR 0.84; 0.42−1.66), forced expiratory volume in one second (OR 0.70; 0.36−1.35) or lung diffusing capacity (OR 1.07; 0.58−1.97).

**Conclusion:**

In the general population, the markedly higher prevalence of breathlessness in women is related to their smaller absolute lung volumes.

## Introduction

Breathlessness is the cardinal symptom of cardiorespiratory disease and is strongly associated with adverse health outcomes.[1, 2] Activity-related breathlessness, measured as a modified Medical Research Council (mMRC) score ≥ 1, is reported by about 25 percent of the general adult population.[3–5] Breathlessness is about twice as common among women than men in the general population, and the reasons are unknown.[3–5] Age, educational level, smoking habits, body mass index (BMI), the presence of comorbidity, and lung function impairment measured as forced expiratory volume in one second (FEV_1_) or forced vital capacity (FVC) in percent of the predicted normal are all associated with the prevalence of breathlessness, but neither of these variables explain the sex difference.[3, 6, 7]

Prevailing but unproven hypotheses are that the disparity in breathlessness between men and women is explained by differences in anxiety or depression, sociocultural differences in symptom report, hormonal changes related to menopause, or that women have smaller airways than men matched for lung size (dysanapsis).[8, 9]

Recent mechanistic studies report that women have a lower maximal ventilatory capacity and use a greater fraction of their ventilatory capacity during exertion. For the same level of work or ventilation, women have increased work of breathing, neural respiratory drive, and exertional breathlessness compared with men.[6, 8, 10–14] The sex difference in breathlessness was attenuated when accounting for differences in absolute ventilatory capacity both in healthy and people with chronic obstructive pulmonary disease (COPD) during standardized exercise in the laboratory,[6, 8, 10–13] and in patients with severe COPD evaluated for lung volume reduction surgery.[15]

However, the mechanistic studies were small, did not evaluate the interplay of multiple factors in a general population, or the importance of the suggested mechanisms for breathlessness related to activities of daily life.[10, 12, 14] A recent population study reported that the higher prevalence of breathlessness in women was related to lower absolute FEV_1_ or FVC, which were used as proxies for absolute lung volume.[5] These findings are yet to be validated. No population study has evaluated breathlessness in daily life in relation to static lung volumes.

The aim of the present study was to evaluate that the increased prevalence of breathlessness in women in the general population is mediated through their lower absolute lung volumes.

## Methods

### Study design and population

This was a cross-sectional analysis of the population-based Swedish CArdioPulmonary bioImage Study (SCAPIS) Pilot. SCAPIS has been detailed elsewhere.[16, 17] The present study recruited a random population sample (n = 1,111) of Swedish residents aged 50 to 64 years in 2012 living in low as well as high socio-economic standard areas in Gothenburg using the Swedish Population Registry. Exclusion criteria were inability to understand written and spoken Swedish for informed consent. This analysis excluded people with difficulties in walking for other reasons than breathlessness (n = 14) and people with missing data on any of the variables specified below (n = 84).

### Data collection and definitions

Self-reported questionnaire data included smoking status, pack years of smoking (number of cigarettes/20/day × years of smoking), menopause (defined as no menstrual bleeding during the past year) and doctor’s diagnoses of asthma, chronic bronchitis and heart disease. Severity of breathlessness was self-reported using the mMRC scale [18] as Grade 0: breathlessness only with strenuous exercise; Grade 1: shortness of breath when hurrying on the level or walking up a slight hill: Grade 2: walking slower than people of the same age on the level because of breathlessness or having to stop for breath when walking on your own pace in the level; Grade 3: stopping for breath after walking about 100 meters or after a few minutes on the level; Grade 4: being too breathless to leave the house or being breathless when dressing or undressing. Anxiety was assessed using the question ‘How often the last 4 weeks have you felt very nervous?’ and categorized as never, or at least sometimes. Depression was defined as self-reported feeling of being sad or depressed for two weeks or more during the recent 12 months together with either loss of usual interest in things or loss of energy. Measurements included weight and height, with body mass index (BMI) calculated as weight(kg)/height(m)^2^, and spirometry including plethysmography was performed using Jaeger Master Screen equipment (Hoechberg, Germany) according to ERS/ATS standards.[19] For FEV_1_ and FVC, post-bronchodilator values 15 minutes after inhalation of 400 μg of salbutamol were used in the analysis. Chronic airflow limitation was defined as FEV_1_/FVC below the 5th percentile (lower limit of normal; LLN). Diffusing lung capacity for carbon monoxide (DLCO) and the DLCO corrected for lung volume (DLCO/V_A_) were measured using a single breath carbon monoxide diffusion test and were adjusted for plasma haemoglobin level.[20] Inspiratory capacity (IC) was calculated as the total lung capacity (TLC) minus the functional residual capacity (FRC). All lung function measures were expressed as absolute values and relative values (percentage of predicted; %pred).[20–24] The extent of dysanapsis was calculated using the formula of Mead et al.[8, 9, 25]

### Statistical analyses

Patient characteristics were tabulated by sex. The association between female sex and higher mMRC breathlessness score were analyzed using ordinal logistic regression, adjusting for age, pack years of smoking, BMI, the presence of chronic airflow limitation, self-reported asthma, chronic bronchitis, heart disease, depression and anxiety.[3–5] mMRC was categorized as 0, 1 and ≥2, categories 2–4 were merged due to low numbers. The proportional odds assumption of the ordinal model was confirmed using multinominal logistic regression, and findings were also robust when the analysis was repeated using a dichotomous logistic model (mMRC ≥ 1 vs 0). The ordinal model was preferred as it used more granular data on mMRC and yielded higher precision compared with other methods. As only few patients with missing data (n = 84), no data were imputed. BMI was included as continuous variable as categorical analysis yielded similar results. The impact of lung function measures on the sex difference in breathlessness was evaluated as the change in the adjusted sex estimate when adding each lung function measure to the adjusted model. Measures of primary interest were TLC, IC, FVC, FEV_1_, DLCO and DLCO/V_A_, as absolute values and %pred. DLCO/V_A_ was analyzed as to delineate whether an association between DLCO and breathlessness was independent of absolute lung volume. Associations were evaluated in men and women separately. We tested whether the association between each lung function variable and breathlessness differed between men and women by including an interaction term in the fully adjusted model. In secondary analyses using the same ordinal regression model and covariates, the relation of height, anxiety, depression, menopause, and dysanapsis to the sex difference in breathlessness were explored.

Associations were expressed as odds ratios (OR) with 95% confidence intervals (CI). Statistical analyses were performed with Stata version 14.2 (StataCorp LP; College Station, TX, USA).

### Ethical considerations

The study was conducted in accordance with the amended Declaration of Helsinki and was approved by the regional ethic committee of Umeå (DNr 2010/228-31) and Gothenburg (DNr 399–16). Written informed consent was provided by all participants.

## Results

Of an initial 1,111 participants, 1,013 participants (91%) had complete data on study variables and were included in the analysis. Participant characteristics are presented in Table 1. Mean age was 58 (standard deviation, 4) years and 49% were women. Absolute lung volumes were lower in women than men, whereas relative lung volumes (%pred) were in the normal range in most people and did not differ by sex (Table 1). The prevalence of mMRC ≥1 was 9% overall, and was twice as high in women (12%) than in men (6%); unadjusted ordinal OR 1.99 (95% CI, 1.28 to 3.11). People with breathlessness (mMRC ≥ 1) also had higher smoking exposure, higher BMI, lower absolute and relative lung function, and higher rates of asthma, chronic bronchitis, anxiety and depression (S1 Table).

**Table 1:** Characteristics of participants.

Patient characteristics	All	Men	Women
N	1,013 (100)	514 (51)	499 (49)
mMRC			
0	920 (91)	481 (94)	439 (88)
1	44 (4)	16 (3)	28 (6)
≥2	49 (5)	17 (3)	32 (6)
Age, y	58 ± 4	58 ± 4	58 ± 4
Smoking status			
Never	434 (43)	211 (41)	223 (45)
Current	182 (18)	89 (17)	93 (19)
Former	397 (39)	214 (42)	183 (37)
Pack years of smoking	10 ± 15	11 ± 15	8 ± 14
Body mass index, kg/m^2^	27.2 ± 4.5	27.7 ± 4.0	26.7 ± 5.0
Hb, mmol/l	140.5 ± 12.3	147 ± 10.7	133.4 ± 9.6
Asthma	94 (9)	39 (8)	55 (11)
Chronic bronchitis	75 (7)	40 (8)	35 (7)
Heart disease	26 (3)	19 (4)	7 (1)
Anxiety			
Never	549 (54)	297 (58)	252 (51)
At least sometimes	464 (46)	217 (42)	247 (49)
Depression	231 (23)	81 (16)	150 (30)
Low socioeconomic status	486 (48)	251 (49)	235 (47)
FEV_1_, L	3.22 ± 0.76	3.71 ± 0.66	2.72 ± 0.47
FEV_1_%pred	101 ± 24	101 ± 24	102 ± 22
FVC, L	4.14 ± 0.96	4.80 ± 0.80	3.46 ± 0.56
FVC%pred	102 ± 24	102 ± 25	101 ± 23
FEV_1_/FVC	0.78 ± 0.07	0.77 ± 0.07	0.79 ± 0.06
FEV_1_/FVC%pred	100 ± 9	99 ± 9	101 ± 8
FEV_1_/FVC < LLN	69 (7)	43 (8)	26 (5)
IC, L	3.10 ± 0.88	3.67 ± 0.75	2.51 ± 0.57
IC%pred	103 ± 21	102 ± 8	104 ± 23
TLC, L	6.34 ± 1.32	7.21 ± 1.10	5.45 ± 0.85
TLC%pred	98 ± 12	97 ± 11	100 ± 13
DLCO, mmol/(min × kPa)	8.27 ± 1.90	9.40 ± 1.73	7.11 ± 1.26
DLCO%pred	96 ± 15	99 ± 16	93 ± 14
DLCO/VA, mmol/(min × kPa × L)	1.49 ± 0.56	1.51 ± 0.67	1.46 ± 0.43
DLCO/VA %pred	94 ± 37	99 ± 45	88 ± 26

Data presented as mean ± standard deviation or frequency (percentage).

*List of abbreviations*: mMRC = modified Medical Research Council breathlessness scale; FEV_1_ = forced expiratory volume in one second; FVC = forced vital capacity; IC = inspiratory capacity; LLN = lower limit of normal; TLC = total lung capacity; DLCO = diffusing lung capacity for carbon monoxide; DLCO/VA = DLCO corrected for lung volume.

In multivariable ordinal logistic regression, the sex difference in breathlessness was OR 2.20 (95% CI, 1.32 to 3.66) for women compared with men. Additional factors that were independently associated with breathlessness were age, number of pack years of smoking, chronic airway limitation, and a comorbid diagnosis of asthma, chronic bronchitis, anxiety and depression (Table 2).

**Table 2:** Factors associated with increased breathlessness in the general population (n = 1,013).

Factor	Breathlessness Odds ratio (95% CI)	P-value
Women vs. men	2.20 (1.32 to 3.66)	0.002
Age (per 1 y)	1.10 (1.04 to 1.17)	0.001
Pack years (per 1 pack year)	1.01 (1.00 to 1.03)	0.049
FEV_1_/FVC < LLN	2.67 (1.27 to 5.60)	0.009
BMI (per 1 kg/m^2^)	1.14 (1.09 to 1.19)	<0.001
Asthma	3.33 (1.85 to 6.01)	<0.001
Chronic bronchitis	2.25 (1.16 to 4.35)	0.016
Heart disease	1.47 (0.46 to 4.67)	0.510
Depression	2.57 (1.54 to 4.28)	<0.001
Anxiety	1.70 (1.01 to 2.87)	0.045

Associations with a higher modified Medical Research Council (mMRC) breathlessness score using multivariable ordinal logistic regression.

*List of abbreviations*: CI = confidence interval; for other abbreviations see Table 1.

**Fig 1:**
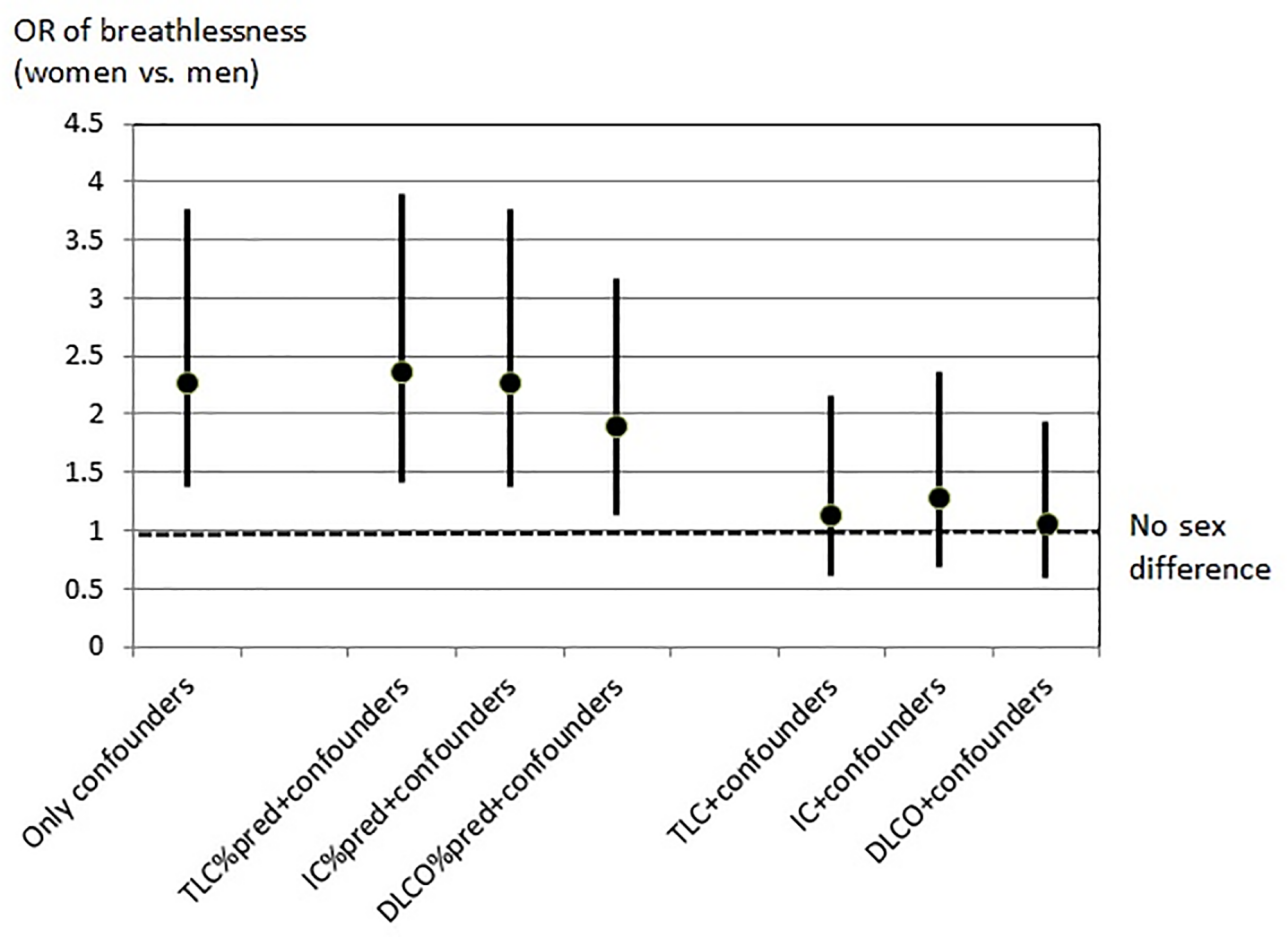
Sex difference in breathlessness. The sex difference is measured as the odd ratio of having a higher modified Medical Research Council (mMRC) score in women compared with men. Estimates are given not adjusting for lung function, and adjusted for relative or absolute lung function, respectively. All models were adjusted for age, pack years, FEV_1_/FVC < lower limit of normal, body mass index, asthma, chronic bronchitis, heart disease, anxiety, and depression. *List of abbreviations*: OR = odds ration; for other abbreviations see Table 1.

Lower absolute lung function was independently associated with increased prevalence of breathlessness both overall and in men and women separately (Table 3). The associations between lung volume and breathlessness were similar in men and women with no signs of interaction (Table 3). The sex difference in breathlessness remained unchanged with further adjustment for relative lung function (%pred) in multivariable analysis (Table 4). In contrast, the sex difference in breathlessness disappeared when adjusting for absolute lung volumes or DLCO (Table 4; Fig 1). As the sex difference remained unchanged when adjusting for DLCO standardized for absolute lung volume (DLCO/V_A_), the effect of absolute DLCO on the sex difference seemed to be mediated through the lung volume (Table 4).

**Table 3:** Associations between absolute lung function and breathlessness adjusted for confounders.

Independent variables	All OR (95%CI)	Men OR (95%CI)	Women OR (95%CI)	P-value for Difference in association between men and women
TLC (per 1 L)	0.65 (0.50 to 0.85)	0.60 (0.39 to 0.91)	0.69 (0.49 to 0.98)	0.50
IC (per 1 L)	0.58 (0.40 to 0.84)	0.59 (0.33 to 1.06)	0.56 (0.34 to 0.90)	0.67
FVC (per 1 L)	0.44 (0.29 to 0.66)	0.40 (0.22 to 0.72)	0.50 (0.28 to 0.90)	0.46
FEV_1_ (per 1 L)	0.27 (1.59 to 0.45)	0.20 (0.09 to 0.44)	0.34 (1.64 to 0.72)	0.35
DLCO (per 1 mmol×min^-1^×kPa^-1^)	0.72 (0.61 to 0.85)	0.69 (0.55 to 0.86)	0.72 (0.56 to 0.93)	0.91
DLCO/V_A_ (per 1 mmol×min^-1^×kPa^-1^×L^-1^)	1.37 (1.03 to 1.82)	1.44 (1.02 to 2.04)	1.30 (0.79 to 2.15)	0.72

Associations of absolute lung function measures with higher modified Medical Research Council (mMRC) breathlessness scores, analyzed using multivariable ordinal logistic regression adjusting for age, pack years, chronic airflow limitation (FEV_1_/FVC < lower limit of normal), body mass index, asthma, chronic bronchitis, heart disease, anxiety, and depression.

*List of abbreviations*: CI = confidence interval; for other abbreviations see Table 1.

**Table 4:** Impact of lung function on the sex difference in breathlessness adjusting for confounders.

Models, all adjusted for confounders	Sex difference in breathlessness, women vs. men, OR (95% CI)	P-value
Not adjusted for lung function, but for confounders	2.20 (1.32 to 3.66)	0.002
**Relative lung function**		
TLC%pred	2.28 (1.37 to 3.80)	0.002
IC%pred	2.19 (1.32 to 3.65)	0.003
FVC%pred	2.25 (1.35 to 3.76)	0.002
FEV_1_%pred	2.30 (1.38 to 3.84)	0.001
DLCO%pred	1.85 (1.10 to 3.10)	0.020
DLCO/V_A_%pred	2.33 (1.39 to 3.91)	0.001
**Absolute lung function**		
TLC	1.12 (0.59 to 2.15)	0.76
IC	1.26 (0.68 to 2.35)	0.47
FVC	0.84 (0.42 to 1.66)	0.61
FEV_1_	0.70 (0.36 to 1.35)	0.28
DLCO	1.07 (0.58 to 1.97)	0.82
DLCO/V_A_	2.26 (1.36 to 3.77)	0.002
**Other factors**		
Menopause	2.26 (1.32 to 3.88)	0.003
Dysanapsis ratio*	2.24 (1.34 to 3.73)	0.002

Odds ratio (OR) of higher modified Medical Research Council (mMRC) breathlessness score in women compared with men, analyzed using multivariable ordinal logistic regression. Each factor was evaluated separately adjusting for the potential confounders: age, pack years of smoking, chronic airflow limitation (FEV_1_/FVC < LLN), BMI, asthma, chronic bronchitis, heart disease, anxiety, and depression.

* Dysanapsis ratio = a measure of decreased airway size relative to lung size, calculated according to Mead et al.[8]

*List of abbreviations*: CI = confidence interval; for other abbreviations see Table 1.

In secondary analyses, being taller was associated with having less breathlessness (OR 0.61; 95% CI, 0.41 to 0.89, per 10 cm increase in height) and this association was similar in women and men. However, when adjusting for any of the absolute lung volume measures, the association of height with breathlessness became non-significant; OR 0.79 (95% CI, 0.50 to 1.25) adjusting for TLC, whereas the association for the lung function parameter remained relative unchanged. The sex difference in breathlessness was not decreased when adjusting for anxiety, depression, menopause or dysanapsis (Table 4). All findings were robust when also adjusting for Hb and socioeconomic status, and when the analyses were repeated using multinominal or dichotomous logistic regression.

## Discussion

The main finding is that the markedly increased prevalence of breathlessness in women was related to differences in absolute lung size. In both men and women, people with smaller lung volumes had higher prevalence of breathlessness. When comparing people with similar absolute lung volume, the prevalence of breathlessness was similar between men and women.

### What this study adds

This is the first population study of sex and breathlessness in relation of absolute lung volumes and diffusion lung capacity. The present study shows that while the sex difference in breathlessness remains with adjustment for relative lung function in %pred, the association disappeared as the estimate approached unity and became non-significant when accounting for absolute lung volumes. This novel finding means that men and women with similar lung size reported similar prevalence of breathlessness. The sex difference was also explained by level of absolute DLCO, which was mediated through absolute lung volumes (smaller lungs have decreased area for diffusion), as DLCO corrected for lung volume (DLCO/V_A_) did not decrease the sex disparity.

The present findings confirm a recent report that the sex difference in breathlessness was related to differences in absolute FEV_1_ and FVC.[5]. Further, the finding of similar associations between lung volumes with breathlessness in both men and women supports the importance of lung size for breathlessness in daily life, as both men and women with smaller lungs were more breathless than their counterparts with larger lungs. The present study also for the first time explored the relation of anxiety, depression, menopause and dysanapsis on the sex difference in breathlessness, and found no evidence that these factors explained the difference in breathlessness between men and women.

### Mechanisms

Compared with men, women have smaller airways and less respiratory musculature, even when matched for height and lung size, resulting in a lower ventilatory capacity [6, 8, 11]. For a given level of work, ventilation or metabolic requirement, women experience more breathlessness than men as a greater fraction of their ventilatory capacity is used.[10, 12, 13] In a laboratory setting, the sex discrepancy in breathlessness was attenuated when accounting for relative maximal ventilatory capacity,[10, 12, 13] which is in line with the present novel findings relating to breathlessness in the general population during daily life.

Breathlessness is a complex sensation caused by the interplay of multiple factors.[26, 27] The present study confirms that several factors are associated with breathlessness in daily life including smoking, BMI, age, and comorbid conditions in terms of chronic airway limitation, chronic bronchitis, asthma, heart disease, depression and anxiety.[3, 6, 7] However, these factors did not explain the sex difference in breathlessness which was mediated through differences in absolute lung volumes.

### Strengths and limitations

Major strengths of the present study are that it included a large population sample with unique data on body plethysmography and DLCO. Effects on breathlessness pertain to people aged 50 to 64 years in the general population and to the symptom’s functional impact assessed using the mMRC scale.[26] Data on standardized exercise tests were not available as exercise tests have limited feasibility in large population-based studies. The mMRC breathlessness scale is highly discriminative and reliable, and is strongly related to important outcomes such as health-related quality of life and mortality in the general population.[2, 28] Data on other dimensions of breathlessness including intensity, unpleasantness, descriptors, and emotional responses were unavailable. In a previous study, differences in the affective descriptors of breathlessness during intensive exercise between men and women were also related to differences in absolute lung volumes.[13] Anxiety and depression were assessed using simple self-rated questions, with no signs that these factors influenced the sex difference in breathlessness, but further study using more multidimensional and validated instruments should be performed. A possible limitation was that the population sample was chosen to represent areas with low as well as high socio-economic standard areas,[16] which could decrease the representativeness for the general population. The lower prevalence of breathlessness compared with previous studies could reflect lower recruitment among people with more severe illness as well as the relatively low smoking exposure in the Swedish population.[3, 4, 7] However, the gender difference in breathlessness was similar to in previous population-based studies [3–5, 7] which supports the generalizability of the findings.

### Implications

The present study supports that the absolute lung volume affects breathlessness in daily life in the general population. When matched on absolute lung volume, there was no sex difference in breathlessness. These findings suggest the importance of evaluating both the relative and the absolute lung volume in research and clinical practice. Relative lung volume reflects the level of lung volume *impairment* compared to the predicted normal, and may inform on an active disease process (such as COPD) that influence the trajectory of lung function decline over time, as well as systemic consequences of the disease, health status, and mortality.[29] The impact of a given lung function impairment on symptoms and function, however, might depend on the person’s absolute lung volume and *remaining ventilatory reserve*. In the present study, a FEV_1_ of 50%pred corresponds to an average FEV_1_ of about 1.35 liters among women, whereas men with the same level of lung function impairment has a mean FEV_1_ of 1.85 liters– 500ml or 37% higher than in women. Although matched on level of lung function impairment, men and women thus may have markedly different absolute lung volumes, which could explain the sex disparity in breathlessness seen in previous studies matching on the FEV_1_%pred.[15, 30–32] An important implication for future clinical studies is that matching on relative lung volume puts women at a disadvantage in relation to breathlessness due to their average lower absolute lung volume. This sex bias can be overcome by accounting for absolute lung volume. In research and clinical practice, absolute lung volume are often not analyzed or reported, and its importance in breathlessness has been largely overlooked. The relation between absolute lung volumes and breathlessness needs to be investigated in people with significant lung function impairment. Another important question which warrants longitudinal analysis is whether people with smaller lungs are at higher risk of developing more severe breathlessness in relation to respiratory disease and noxious exposures including tobacco smoke and environmental pollution. People with smaller lung volumes might be a risk group that warrants closer evaluation, intensified efforts to prevent lung function impairment, and closer follow-up. Relative and absolute lung volumes provide complementary information on the lung volume impairment and remaining ventilatory reserve and should be evaluated in both research and clinical care.

## Supporting information

S1 Table Characteristics of people without or with breathlessness. Data presented as mean ± standard deviation or frequency (percentage). *List of abbreviations*: mMRC = modified Medical Research Council breathlessness scale; FEV_1_ = forced expiratory volume in one second; FVC = forced vital capacity; IC = inspiratory capacity; LLN = lower limit of normal; TLC = total lung capacity; DLCO = diffusing lung capacity for carbon monoxide; DLCO/VA = DLCO corrected for lung volume.
(DOCX)

## Abbreviations

BMI: body mass index
CI: confidence interval
COPD: chronic obstructive pulmonary disease
DLCO: diffusing lung capacity for carbon monoxide
DLCO/V_A_: diffusing lung capacity for carbon monoxide corrected for lung volume
FEV_1_: forced expiratory volume in one second
FRC: functional residual capacity
FVC: forced vital capacity
IC: inspiratory capacity
LLN: lower limit of normality
mMRC: modified Medical Research Council breathlessness score
OR: odds ratio
SCAPIS: Swedish CArdioPulmonary bioImage Study
TLC: total lung capacity
