# Supplementary material for: Absolute lung size and the sex difference in breathlessness in the general population
Source: PLoS One. 2018 Jan 5;13(1):e0190876. doi: 10.1371/journal.pone.0190876 (PMC5755925; doi:10.1371/journal.pone.0190876)
Supplement: S1 Table — Data presented as mean ± standard deviation or frequency (percentage). List of abbreviations: mMRC = modified Medical Research Council breathlessness scale; FEV1 = forced expiratory volume in one second; FVC = forced vital capacity; IC = inspiratory capacity; LLN = lower limit of normal; TLC = total lung capacity; DLCO = diffusing lung capacity for carbon monoxide; DLCO/VA = DLCO corrected for lung volume. (DOCX) [file pone.0190876.s001.docx]

**S1 Table. Characteristics of people without or with breathlessness**

| **Patient characteristics** | **mMRC 0** | **mMRC ≥ 1** |
| --- | --- | --- |
| N | 920 (91) | 93 (9) |
| Men | 481 (52) | 33 (35) |
| Age, y | 58 ± 4 | 59 ± 4 |
| Smoking status  Never  Former  Current | 406 (44)  159 (17)  355 (39) | 28 (30)  23 (25)  42 (45) |
| Pack years of smoking | 9 ± 14 | 15 ± 22 |
| Body mass index, kg/m^2^ | 26.9 ± 4.2 | 30.4 ± 6.0 |
| FEV_1_/FVC < LLN | 52 (6) | 17 (18) |
| Asthma | 69 (8) | 25 (27) |
| Chronic bronchitis | 57 (6) | 18 (19) |
| Heart disease | 21 (2) | 5 (5) |
| Anxiety  Never  At least sometimes | 517 (56)  403 (44) | 32 (34)  61 (66) |
| Depression | 185 (20) | 46 (49) |
| FEV_1_, L  FEV_1_%pred | 3.29 ± 0.75  103 ± 23 | 2.60 ± 0.62  85 ± 24 |
| FVC, L  FVC%pred | 4.21 ± 0.96  103 ± 24 | 3.51 ± 0.81  89 ± 25 |
| FEV_1_/FVC  FEV_1_/FVC%pred | 0.78 ± 0.06  100 ± 8 | 0.75 ± 0.1  96 ± 13 |
| IC, L  IC%pred | 3.14 ± 0.88  104 ± 20 | 2.71 ± 0.79  91 ± 22 |
| TLC, L  TLC%pred | 6.41 ± 1.31  99 ± 11 | 5.65 ± 1.27  97 ± 16 |
| DLCO, mmol/(min×kPa)  DLCO%pred | 8.38 ± 1.88  97 ± 15 | 7.16 ± 1.72  89 ± 17 |
| DLCO/V_A_, mmol/(min×kPa×L)  DLCO/V_A_ %pred | 1.48 ± 0.53  93 ± 35 | 1.57 ± 0.81  96 ± 52 |

Data presented as mean ± standard deviation or frequency (percentage).

*List of abbreviations:* mMRC = modified Medical Research Council breathlessness scale; FEV_1_ = forced expiratory volume in one second; FVC = forced vital capacity; IC = inspiratory capacity; LLN = lower limit of normal; TLC = total lung capacity; DLCO = diffusing lung capacity for carbon monoxide; DLCO/VA = DLCO corrected for lung volume.
